# The tumor promoter‐activated protein kinase Cs are a system for regulating filopodia

**DOI:** 10.1002/cm.21373

**Published:** 2017-05-24

**Authors:** Carol A. Heckman, Pratima Pandey, Marilyn L. Cayer, Tania Biswas, Zhong‐Yin Zhang, Nancy S. Boudreau

**Affiliations:** ^1^ Department of Biological Sciences Bowling Green State University Life Sciences Building Room 217 Bowling Green Ohio 43403; ^2^ Center for Microscopy and Microanalysis Bowling Green State University Life Sciences Building Room 217 Bowling Green Ohio 43403; ^3^ Department of Medicinal Chemistry and Molecular Pharmacology Purdue University Robert E. Heine Pharmacy Building, Room 202A, 575 Stadium Mall Drive West Lafayette Indiana 47907; ^4^ Department of Applied Statistics and Operations Research Bowling Green State University 344 Business Administration Building Bowling Green Ohio 43403

**Keywords:** adhesion, cancer, contact inhibition, protrusions, signaling

## Abstract

Different protein kinase C (PKC) isoforms have distinct roles in regulating cell functions. The conventional (α, β, γ) and novel (δ, ɛ, η, θ) classes are targets of phorbol ester tumor promoters, which are surrogates of endogenous second messenger, diacylglycerol. The promoter‐stimulated disappearance of filopodia was investigated by use of blocking peptides (BPs) that inhibit PKC maturation and/or docking. Filopodia were partially rescued by a peptide representing PKC ɛ hydrophobic sequence, but also by a myristoylated PKC α/β pseudosubstrate sequence, and an inhibitor of T‐cell protein tyrosine phosphatase (TC‐PTP). The ability to turn over filopodia was widely distributed among PKC isoforms. PKC α and η hydrophobic sequences enhanced filopodia in cells in the absence of tumor promoter treatment. With transcriptional knockdown of PKC α, the content of PKC ɛ predominated over other isoforms. PKC ɛ could decrease filopodia significantly in promoter‐treated cells, and this was attributed to ruffling. The presence of PKC α counteracted the PKC ɛ‐mediated enhancement of ruffling. The results showed that there were two mechanisms of filopodia downregulation. One operated in the steady‐state and relied on PKC α and η. The other was stimulated by tumor promoters and relied on PKC ɛ. Cycles of protrusion and retraction are characteristic of filopodia and are essential for the cell to orient itself during chemotaxis and haptotaxis. By suppressing filopodia, PKC ɛ can create a long‐term “memory” of an environmental signal that may act in nature as a mnemonic device to mark the direction of a repulsive signal.

## 1 INTRODUCTION

There are 10 enzymes in the PKC family, and eight are targets of tumor promoters. Despite the time elapsed since 1982, when it was first reported that tumor promoters activate PKC, the way in which the PKCs promote tumors remains obscure. All isoforms have an N‐terminal half with a number of regulatory domains and a C‐terminal portion housing the active enzyme. All have a phosphatidylserine‐binding domain near the N‐terminal that localizes them to membranes. The conventional PKCs (cPKCs) have calcium‐binding domains that are missing from the novel protein kinase Cs (nPKCs), but both classes bind the endogenous activator, diacylglycerol. A third class, called atypical isoforms, is not activated by phorbol esters or diacyglycerol. The α and δ isoforms, along with atypical isoform, λ, are ubiquitous, but the remaining isoforms are expressed in different combinations in different cell types.

PKCs are spatially restricted by binding to scaffolds called receptors for activated C kinases (RACKs). These include RACK1, which probably binds most PKC isoforms (Besson, Wilson, & Yong, 2002; Korchak and Kilpatrick, 2001), [reviewed in (Adams, Ron, & Kiely, 2011], and RACK2/β′‐COP, a coated vesicle protein that binds activated nPKCs. RACK1 is involved in the regulation of the actin cytoskeleton and filopodia formation (Demarco and Lundquist, 2010). RACK2 is involved in vesicle trafficking and cell‐cell communication [reviewed in (Schechtman and Mochly‐Rosen, 2001)]. PKC ɛ has long been known to be localized to Golgi apparatus by RACK2 (Csukai, Chen, De Matteis, & Mochly‐Rosen, 1997; Peterson and Stamnes, 2013), specifically by an amino acid sequence, 14–21, in the extreme N‐terminus of the protein (Yedovitzky et al., 1997) and phosphoserine 729 in the extreme C‐terminus (Xu, He, Dobson, England, & Rumsby, 2007). Restricting the location of PKCs may put constraints on their access to agonists, especially diacylglycerol (Almena and Mérida, 2011). There are additional biophysical and biochemical mechanisms that keep the enzymes from being inappropriately activated. At the N‐terminal portion of the protein, there is a pseudosubstrate sequence, which binds tightly to the active site until displaced by an agonist. Second, when activated in membranes, the PKCs become susceptible to hydrolysis by two types of calpains. The C‐terminal half is further degraded by a calpain isozyme that is activated at high calcium levels (Cressman, Mohan, Nixon, & Shea, 1995), [reviewed in (Franco and Huttenlocher, 2005; Steinberg, 2008)].

PKCs undergo a maturation process in which the molecules become competent or “primed” through phosphorylation at multiple sites. Phosphate addition at a site in the activation loop is essential for maturation to the competent form and is preceded or followed by phosphorylation of two sites near the C‐terminal end of the molecule (Keranen, Dutil, & Newton, 1995; Lachmann, Bär, Rommelaere, & Nüesch, 2008), [reviewed in (Griner and Kazanietz, 2007)]. Phosphate addition to these sites, called the “turn” and “hydrophobic” motifs, is required for the maturation and stabilization of the enzyme (Bornancin and Parker, 1996; Ikenoue, Inoki, Yang, Zhou, & Guan, 2008). The kinase, 3‐phosphoinositide‐dependent protein kinase 1 (PDK1) is thought to be responsible for activation loop phosphorylation (Frödin, Jensen, Merienne, & Gammeltoft, 2000; Gao, Toker, & Newton, 2001; Newton, 2003). However, for PDK1 to dock on PKC, a negative charge on the hydrophobic motif is required (Balendran et al., 2000; Frödin et al., 2000) as well as anchorage in a membrane (Cenni et al., 2002). Certain endogenous isoforms are found to be poor in phosphorylation at this site, including PKC η (Lachmann et al., 2008). Although activation loop phosphorylation is sometimes considered a marker of PKC maturation, this site can be dephosphorylated with retention of enzymatic competency as long as the turn and hydrophobic sites of phosphorylation are occupied. Moreover, phosphorylation or other modifications elsewhere on the molecule may also be important for folding of PKC into a ‘closed’ and stable conformation (Freeley, Kelleher, & Long, 2011).

Phosphorylation at the hydrophobic motif also creates a site for docking the downstream effector phospholipase D1 (PLD1), which is activated by binding PKC (Hu and Exton, 2003). Consistent with PKC's importance in signal transduction, additional regulatory constraints affect its recycling and degradation. Binding of a substrate or even an inhibitor to the active site prevents dephosphorylation of one or both C‐terminal sites and thereby stabilizes the enzyme (Gould et al., 2011), and the same is true for protein kinase D (PKD), also known as PKC µ (Kunkel and Newton, 2015). For the cPKC isoforms, phosphate additions at the “turn” and “hydrophobic” motifs are controlled by the mammalian target of rapamycin complex (mTORC), which regulates many aspects of protein synthesis (Facchinetti et al., 2008). Alternatives have been suggested for the kinase mediating phosphorylation of the hydrophobic motif, and many different kinases may add phosphate at this site (Dong and Liu, 2005). Furthermore, regulation of PKC phosphorylation and dephosphorylation shows specificity at the isoform and signaling pathway level, as well as cell‐type specificity (Freeley et al., 2011). Finally, some PKCs in their mature form are homodimers. Dimeric conformations are thought to have a prolonged residence time in membranes (Swanson et al., 2014).

The cell transmits communications from the extracellular environment in part by creating diacylglycerol. The phorbol ester tumor promoters, such as phorbol 12‐myristate 13‐acetate (PMA), act as surrogates of diacylglycerol. Both agonists activate PKCs and translocate them to membranes. A sustained elevation of diacylglycerol levels is sometimes produced from a phosphatidic acid precursor, and the site of production can be localized to one type of subcellular membrane to affect cellular functioning (Almena and Mérida, 2011; Liu and Heckman, 1998). Thus, each PKC isoform may be activated at a different site, and there the enzyme may phosphorylate and modify substrates that, in turn, promote or prohibit its access to other compartments. This is how the effects of PKC signaling are orchestrated within the superstructure of the cell. Specifically, this type of dynamic interplay underlies mechanisms by which vesicle trafficking (Kiley, Jaken, Whelan, & Parker, 1995), chemotactic activity (Daviet, Herbert, & Maffrand, 1990), and signal transduction are regulated by PKC. For example, using low PMA levels similar to those we use here, Rehder's group showed that filopodia responded rapidly to phorbol ester, becoming shorter and fewer (Bonsall and Rehder, 1999). The opposite was found by other workers who found that a PKC inhibitor, GF 109203X, caused rapid retraction of growth cone filopodia (Fagerstrom, Påhlman, Gestblom, & Nanberg, 1996). Such discrepancies may be due to differences in the number of PKC isoforms, their varied patterns of distribution in different cell types, and/or the large number of substrates they phosphorylate. From one cell type to another, a single isoform can take on a different role in modulating cytoskeletal and adhesive structures.

The same variations have been impediments to understanding PKC's role in tumor promotion—a process which allows the emergence of a tumor from a cell population that was exposed to a small, initiating dose of carcinogen. Despite the importance of tumor promotion as a mechanism in cancer development, it has resisted scientists' attempts to explain it based on elementary regulatory features of the PKCs. By deconstructing the effects of PMA on cell phenotypes, we hope to identify the targets of activated PKC that are relevant to tumor promotion. We showed previously that filopodia declined immediately after PMA exposure (Heckman, Varghese, Cayer, & Boudreau, 2012). Stress fibers, also called actin cables, underwent a decrease briefly in PMA‐treated cells but then achieved a higher steady‐state level at 5 h (Li, Urban, Cayer, Plummer, & Heckman, 2006). Because stress fibers and filopodia both depend on actin bundles to form an inflexible rod‐like structure at their core, the current research was carried out to investigate the relationship of the PMA‐activated isoforms to the filopodia. Our methods enable the phenotype to be classified on the basis of irreducible features that arise organically from the arrangement of the cell's parts. This type of feature classification removes the difficulty of subjective decision‐making and provides information that may be valuable in understanding tumor promotion. Moreover, we use a cell line called 1000W that was developed as a model of human bronchogenic carcinoma. Its features were defined by unbiased classification methods, so that they could be related to the time course of tumor promotion (see Materials and Methods).

Filopodia act as antennae for incoming signals and are required for cells to compare the strength of adhesion from opposite directions on a substrate (Amarachintha et al., 2015). In an analysis of cancer development, we found that the loss of filopodia accounted for a greater proportion of the quantifiable morphological changes than any other feature (Heckman and Jamasbi, 1999). Filopodia typically undergo cyclical protrusion and retraction, however, and each protrusion‐retraction cycle is regulated by the rates of actin assembly and actin rearward flow. In an analysis of these rates for the nerve cell growth cone, where the filopodia are large and numerous, previous workers found that the rate of rearward flow and actin depolymerization was constant. Thus, filament disassembly at the base sufficed to ensure that retraction occurred if the rate of actin assembly at the tip of the filopodium failed to keep up with disassembly (Mallavarapu and Mitchison, 1999). The current research reveals that filopodia prevalence is regulated by PKC in two ways. The first is a promoter‐mediated mechanism that decreases filopodia, and the second is a steady‐state mechanism that regulates prevalence but is not responsive to activation of PKCs by tumor promoter.

## 2 RESULTS

### 2.1 Actin architectural features regulated by PKC

**Figure 1:**
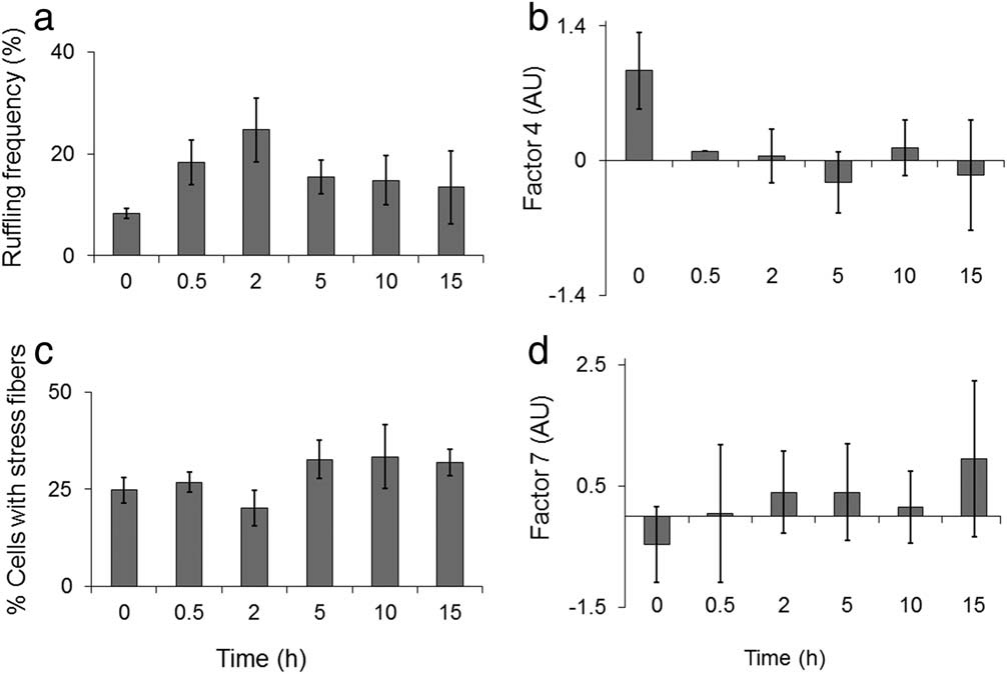
Different actin‐based architectures of 1000W cells show different patterns of prevalence after PMA exposure. (a) Ruffling frequency, (b) Filopodia as measured by factor 4 values, (c) Stress fibers, (d) Neurites as measured by factor 7 values. Means of 2–6 replicated experiments are shown. Factor 4 and 7 values are given in arbitrary units (AU). Statistics on ruffling activity, filopodia, stress fibers, and neurites respectively are shown elsewhere (Heckman et al., 1996, 2004, 2012; Li et al. 2006). Bars represent ± 1 standard error of the mean (SEM).

**Figure 2:**
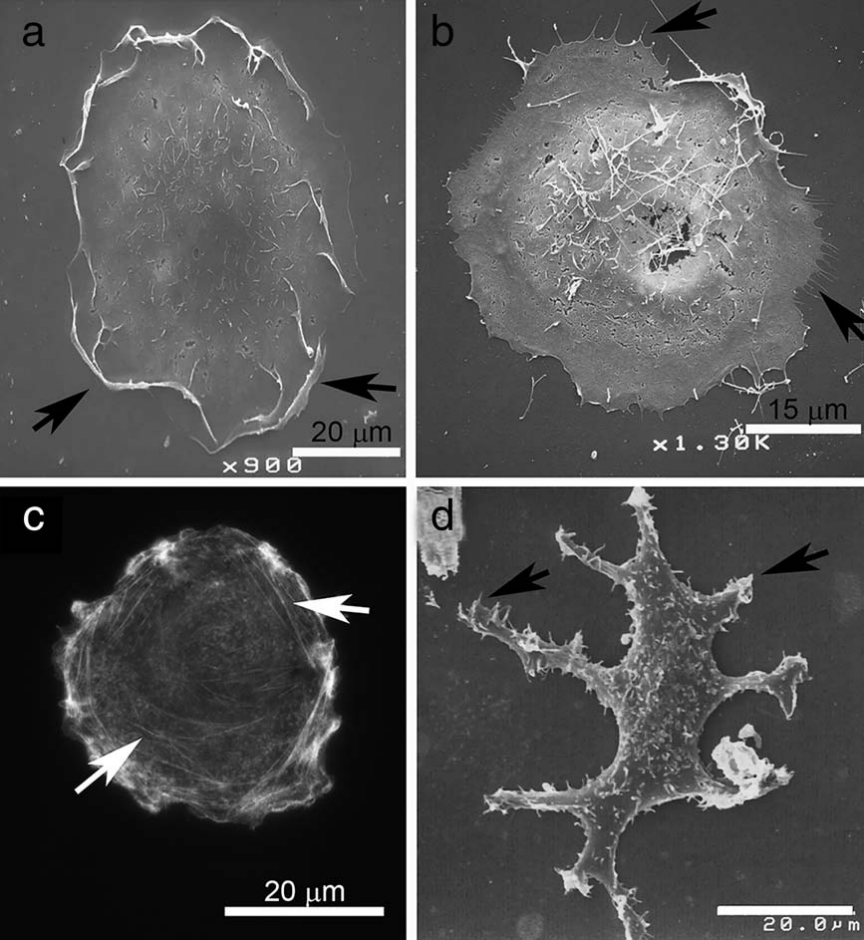
Typical appearance of the actin architectures referred to in Figure 1. (a) Ruffles, (b) Filopodia, (c) Stress fibers, (d) Neurites. Images are made by scanning electron microscopy, except for frame C. In frame C, actin is stained by tetrarhodamine phalloidin and viewed by epifluorescence microscopy. The features are indicated by arrows

Different actin‐based protrusions are assembled and disassembled at different times after treatment of the cell culture with PMA. Ruffles and filopodia showed the most immediate responses. Whereas ruffling activity increased for 2 h and then declined, filopodia declined very rapidly and never recovered over the 15‐h time course (Figure 1a,b). Stress fibers, also called actin cables, decreased briefly and then achieved a higher steady‐state level at 5 h (Figure 1c). Neurites increased gradually throughout the entire time course (Figure 1d). In these experiments, filopodia and neurites were measured by an objective method that obviated the problem of observer bias (see Materials and Methods). Figure 2 illustrates the appearance of each type of feature. Although the features' precise numerical values were not reproduced from one experiment to another, the pattern of change of each feature after exposure to tumor promoter was reproducible. Thus, there was reason to think that the prevalence of the features could be measured with some reproducibility after exposing cultured cells to different reagents.

It was possible to explain the data of Figure 1c as a function of actin bundle disassembly, as shown in previous studies. Stress fibers were negatively regulated by PKC ɛ, which was greatly reduced in 1000W cells by PMA‐initiated activation and subsequent degradation (Li et al. 2006). The role of PKC ɛ and the mechanism of degradation are depicted graphically in Supporting Information Figures S1A and S2A, respectively. PMA affected filopodia much faster than stress fibers, but it is possible that their actin bundles were similarly affected by PKC activation. For example, PKC could be recruited to substrates in the actin core of the filopodia, which would then be rendered susceptible to dissolution after PMA. To determine whether this effect was mediated by PKC, we treated cells with agents that interfered separately and selectively with enzyme maturation and activation, according to the scheme shown in Supporting Information Figure S1B. To inhibit maturation, we used blocking peptides (BPs) that competed for the phosphorylation site at the hydrophobic motif. Here, the effect would be apparent after a long exposure to the agents. In addition to PKC maturation, the BP may block enzyme localization to a scaffold (see Introduction). Then, short‐term exposure to PMA was used to determine whether the filopodia were still disappearing rapidly after PMA treatment.

### 2.2 PKC α and η normally turn over filopodia but PKC ɛ turns them over in response to PMA

**Figure 3:**
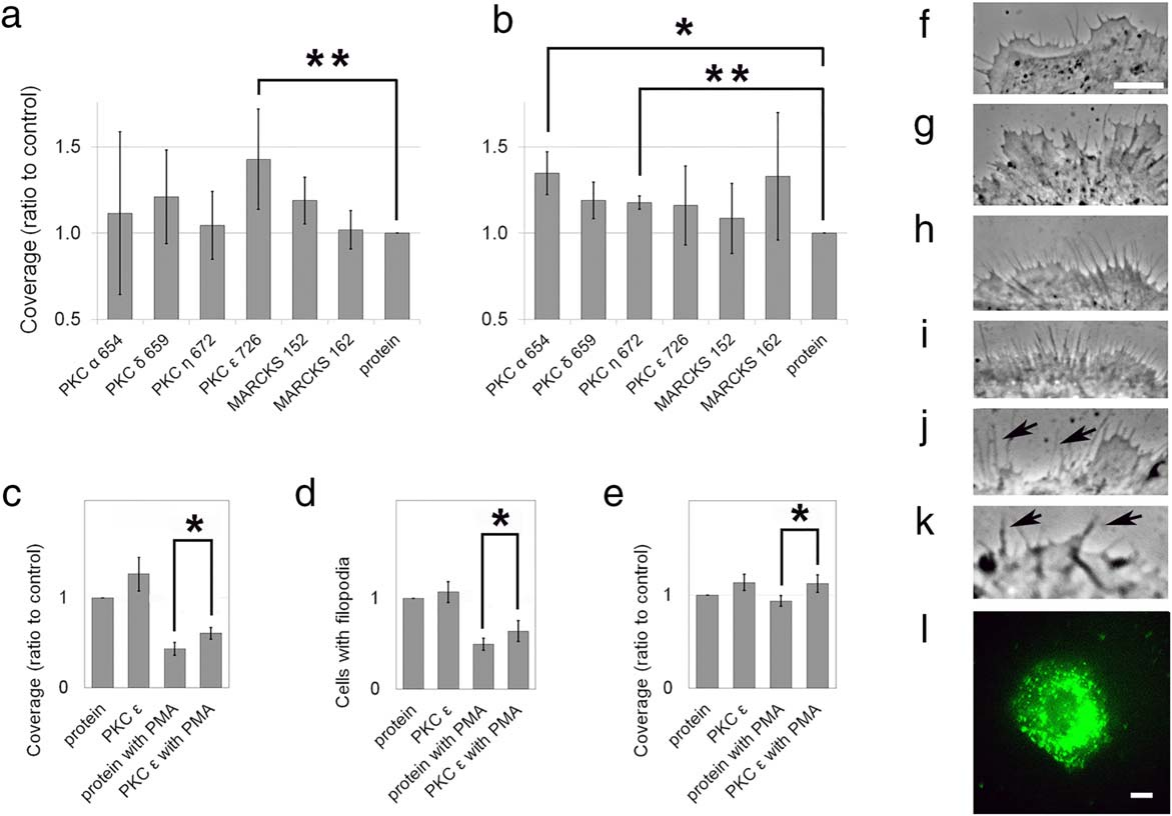
Prevalence of filopodia in 1000W cells treated with BPs representing PKC hydrophobic segments or MARCKS phosphorylation sites. (a) Coverage in BP‐treated samples compared to value of the PMA‐treated control. (b) Coverage in BP‐treated samples without PMA treatment compared to value of the sham‐treated control. (c) Percent coverage of the perimeter with PKC ɛ BP with and without PMA compared to control. (d) Fraction of cells showing filopodia with PKC ɛ BP with and without PMA compared to control. (e) Percent coverage, calculated only for cells showing filopodia, compared to control. For frames a–e, bars represent the mean ± 1 SEM. *difference significant at *p* < 0.05, **difference significant at *p* < 0.025. For (a) and (b), *n* = 4. For frames (c–e), *n* = 5 for PKC ɛ without PMA; *n* = 7 for control containing an irrelevant protein with PMA; *n* = 6 for PKC ɛ with PMA. (f–i) Appearance of filopodia in sham‐treated control and samples that show increases over respective controls, (f) control, (g) PKC α without PMA, (h) PKC ɛ with PMA, (i) PKC η without PMA. (j) Portion of cell edge illustrating retracting filopodia (arrows). (k) Portion of cell edge showing protrusions too large to be considered filopodia (arrows). (l) Pattern of internalization of the irrelevant protein with BioPORTER reagent imaged by epifluorescence microscopy (Amarachintha et al., 2015). Bar = 10 µm [Color figure can be viewed at wileyonlinelibrary.com]

**Figure 4:**
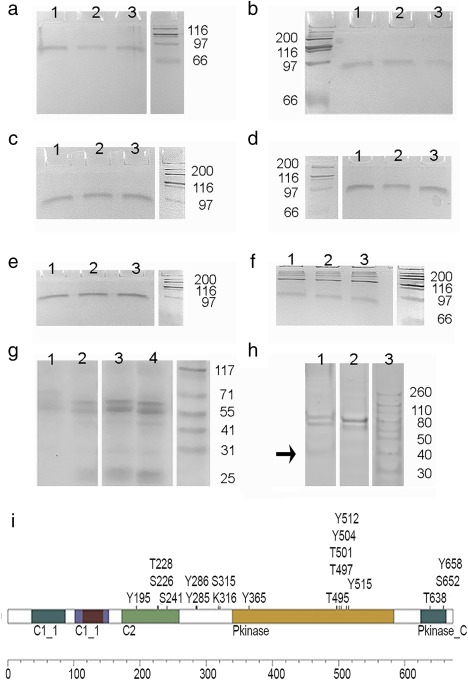
PKC isoforms in 1000W cells over 0–2 h of PMA exposure. (a) PKC α, (b) PKC β, (c) PKC γ, (d) PKC δ, (e) PKC ɛ, (f) PKC η, (g–i) PKC α. PKCs were recovered by immunoprecipitation (see Materials and Methods). (a–f) PKCs recovered under denaturing conditions. Lanes 1, 2, and 3 recovered at times of 0 h, one‐half hour and 2 h respectively. (g) PKC α recovered under non‐denaturing conditions with phospho‐PKC α (Ser657/Tyr658) antibody. Lanes 1 and 3, recovery from 30 to 120 µL of lysate after 1‐h treatment with solvent vehicle. Lanes 2 and 4, recovery from 30 µl or 120 µL of lysate after 1‐h treatment with PMA. (h) PKC α recovery under denaturing conditions from cells treated with PKC α BP or irrelevant protein. Lane 1 = sample treated with PKC α BP. Lane 2 = sample treated with irrelevant protein. A degradation product containing the extreme C‐terminal is indicated by the arrow. (i) Experimentally confirmed phosphorylation sites in PKC α, adapted from http://www.phosphosite.org [Color figure can be viewed at wileyonlinelibrary.com]

To determine whether the BP rescued the filopodia from destruction by PMA, we analyzed the coverage of the cell perimeter with filopodia in replicate samples following introduction of a hydrophobic segment specific for each of the cPKCs and nPKCs. We also analyzed the effects of BPs representing phosphorylation sites of the myristoylated alanine‐rich C kinase substrate (MARCKS), a protein thought to be a universal substrate of PKC. The greatest effect was exerted by the BP for PKC ɛ, as indicated by a 40% elevation in the coverage of the perimeter (Figure 3a). The difference was significant in the one‐tailed *t* test at a level of probability, *p* < 0.025. BPs representing the homologous PKC δ sequence and the phosphorylation site in MARCKS 152–162 also caused an elevation over control, but these results were not statistically significant. The finding that PKC ɛ BP is directly engaged in filopodia destruction was not surprising, because PKC ɛ had caused dissociation of actin cables in PMA‐treated cells (Li et al. 2006). To determine whether the PKCs were downregulated in PMA‐treated cells, accounting for the PMA‐dependent difference in filopodia, we determined the content of the isoforms. We found no difference over times of 0–2 h treatment (Figure 4a‐f). This was probably because the levels of PMA delivered to cell cultures in the current experiments were very low. Over periods of time, two isoforms, PKC γ and PKC ɛ, were degraded (see Supporting Information Figure S2B,C).

In the absence of PMA, BPs representing the PKC α and η sequences significantly increased the percentage of the perimeter covered by filopodia (Figure 3b). As there was no exogenous activator such as PMA in the cells, these increases reflected regulation in the steady state. Moreover, since the BPs conserved filopodia, the results implied that the PKCs inhibit filopodia in the steady state. Because PKC α is activated synergistically by calcium and diacylglycerol in nature, it may not have been activated by PMA under conditions used in the experiments. The PKC α BP had no net effect on filopodia in PMA‐treated cells, but there was extreme variability in the PKC α BP experimental results (Figure 3a). The hydrophobic motif residues targeted by the BP were serine 657 and tyrosine 658. To determine whether they were phosphorylated in 1000W cells, we recovered PKC α under non‐denaturing conditions, using an antibody specific to phospho‐S657/Y658. Under these conditions, PKC was cleaved and the C‐terminal portion of the molecule was recovered as double or triple bands (Figure 4g). This result was similar to those obtained by other laboratories using antibodies against dephosphorylated (England and Rumsby, 2000; Nadra et al., 2005) or phosphorylated (England and Rumsby, 2000; Mirandola et al., 2006) portions of PKC. The PKC α recovery with anti‐phospho‐S657/Y658 was ∼45% greater after 1‐h PMA treatment. This suggested that phosphorylation at the hydrophobic motif was enhanced after PMA treatment because of changes in recycling or maturation. For technical reasons, it is not possible to conclude that PKC α was specifically affected by PMA in the treated cells (see Materials and Methods). Although antibody against phospho‐S657/Y658 was unlikely to recognize the dephosphorylated motif (Rybin and Steinberg, 2006), the antibody may also recover PKC β and other PKC isoforms. Nevertheless, processing at the hydrophobic site was enhanced by PMA treatment for one or more isoforms.

To determine the effect of BP introduction, we used sodium dodecylsulfate‐polyacrylamide gel electrophoresis (SDS‐PAGE) to evaluate proteins from samples treated by PKC α BP or an irrelevant protein. Under denaturing conditions, an antibody directed against the extreme C‐terminal sequence, which is rarely phosphorylated in living cells, recovered a full‐length doublet when phosphatase inhibitors were included in the lysis solution (Figure 4h,i). A protein degradation product appeared at ∼37 kDa in PKC α recovered from the BP‐treated sample, consistent with the known increase in degradation rate of dephosphorylated PKC (Gysin and Imber, 1996). As similar amounts of PKC α were recovered with antibodies against the dephosphorylated region around S670 and with phospho‐S657/Y658 antibody, a fraction of the PKC α molecules in 1000‐W cells may be dephosphorylated at S657. The origin of doublets, such as appeared in the PKC α samples, was investigated further by an immunoprecipitation technique. Phosphorylated molecules bind less SDS and are often less mobile than their dephosphorylated counterparts (Lee, Park, Kim, Peterkofsky, & Seok, 2013). Doublets were recovered under denaturing conditions from PKC α when a tyrosine phosphatase inhibitor, orthovanadate, was included in the lysis solution (see Supporting Information Figure S2E). Under non‐denaturing conditions, PKC ɛ recovered with an antibody against dephosphorylated C‐terminal sequence also exhibited a doublet (data not shown). Although these bands suggest multiple phosphorylation states, other modifications such as acetylation, ubiqitylation, or sumoylation could be responsible for the different mobility on SDS‐PAGE (Freeley et al., 2011). Because of the numerous combinations of specific sites of phosphate addition on the PKC isoforms (Figure 4i), it is invalid to infer activity from the presence or absence of phosphate residues at the activation loop (Kunkel and Newton, 2015; Rybin and Steinberg, 2006). Nevertheless, the data suggest that some of the PKC α is phosphorylated at the hydrophobic motif in the steady‐state, and some molecules undergo further modification during short‐term PMA treatment, which may take the form of phosphate addition.

BPs for PKC β and γ were also studied in a few experiments. The PKC β hydrophobic motif appeared to have no effect when interference with filopodia was analyzed. With the PKC γ BP, the cells developed large, tapering protrusions that could be confused with filopodia. This effect made the samples difficult to evaluate, but it was observed when either BP or transcriptional knockdown agents were employed. Samples treated with BPs for phosphorylation sites in MARCKS and PKC δ showed no significant difference compared to controls (Figure 3a,b).

It should be noted that the percentage of the perimeter covered by filopodia was a combination of two other measures. One was the fraction of cells capable of making filopodia and the second, the density of these protrusions on those cells that made filopodia. To determine whether both were affected, we expressed the results for the underlying variables separately. We also included samples from additional experiments in which the PKC ɛ BP was tested along with other reagents, so there were more experiments to compare. Coverage values again showed significance when compared to PMA‐treated control (cf. Figure 3a,c). The difference between the PMA‐treated samples with and without PKC ɛ BP was significant for the fraction of cells showing filopodia (Figure 3d). Likewise, when the coverage was calculated using only those cells showing filopodia as the denominator, the results were significant (Figure 3e). Thus, PKC ɛ BP affected both the cells' ability to initiate or maintain filopodia and the number of filopodia on the cells that could make them. Filopodia were unaffected by PKC ɛ peptide treatment in the absence of PMA. The “rescue” by BP suggested that filopodia turnover by the tumor promoter had a specific reliance on PKC ɛ. For treatments that increased coverage, we recorded the appearance of the filopodia. Slight differences were seen, but no distinct phenotypes could be recognized as being dependent on a specific isoform (Figure 3f–i). Structures that were excluded from the analysis are shown: (1) filopodia thought to be retracting, indicated by an increase in flexibility (Figure 3j), and (2) structures too large to fit the definition of filopodia (Figure 3k). The pattern of BP internalization with BioPORTER reagent was investigated by imaging the distribution of the irrelevant protein used as control. The protein was found in the Golgi region and diffusely throughout the cytoplasm (Figure 3l).

It was surprising that BPs enhanced filopodia in the absence of PMA, and perhaps even more surprising, that these BP sequences represented both the cPKC and nPKC classes. In comparing the effects of BPs directed against PKC ɛ and η, however, it should be noted that activation was detected only for the isoforms ɛ and γ under the current experimental conditions (Supporting Information Figure S2). It is possible that PKC η failed to destroy filopodia because its activity was not affected by PMA under the conditions of the experiments. Previous workers also found PKC η to be resistant to conformational changes after PMA exposure (Kang, French, Sando, & Hahn, 2000).

### 2.3 A PKC inhibitor reproduces the pattern of filopodia rescue

**Figure 5:**
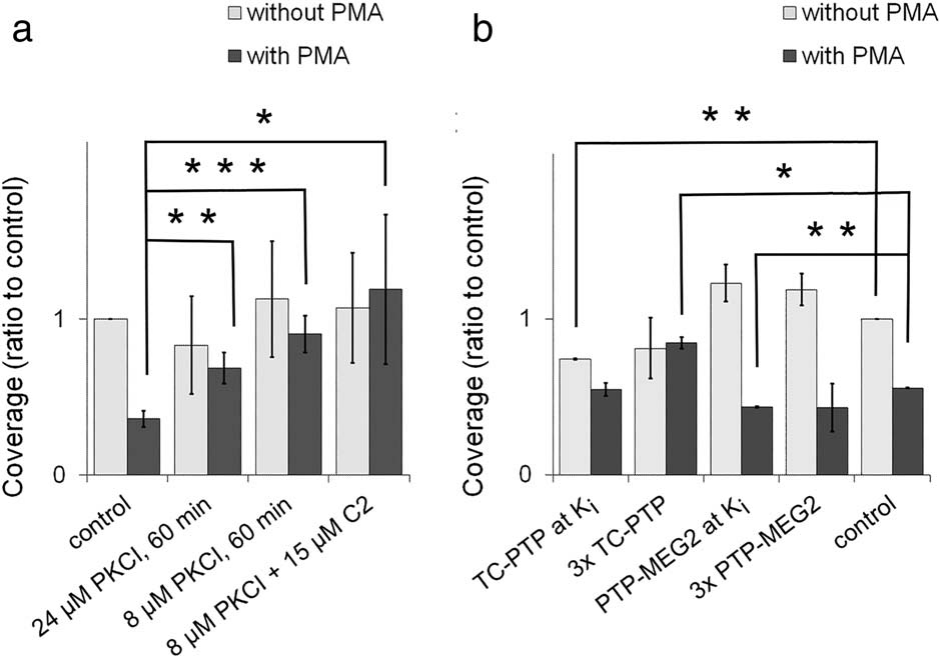
Filopodia coverage in 1000W populations treated with small molecule inhibitors. (a) Effect of PKC α/β inhibitor at two concentrations with and without PMA and C2‐ceramide. The mean is shown ± 1 SEM. For 24 µM concentration with and without PMA, *n* = 3. For 8 µM concentration with and without PMA, *n* = 5 and *n* = 3, respectively. For 8 µM with C2‐ceramide, with or without PMA, *n* = 4. (b) Effect of specific inhibitors of TC‐PTP and PTP‐MEG2 with and without PMA. The mean of two experiments is shown ± 1 SEM. *difference significant at *p* < 0.05, **difference significant at *p* < 0.025, *** difference significant at *p* < 0.001. When combined data for two concentrations of PTP‐MEG2 were tested against control, the difference was significant at *p* < 0.004

To clarify these findings, we introduced a PKC inhibitor (PKCI, Myr19–27) representing the myristoylated nonapeptide pseudosubstrate sequence of PKC α/β. The sequence of this peptide was identical to PKC α/β and a near match for the PKC γ sequence and may be a competitive inhibitor of all PKCs. Treatment with a high concentration, which had inhibited PLD‐dependent physiological effects in previous studies, showed variable effects and was often inhibitory (Figure 5A). At a low concentration, representing the IC50 for MARCKS phosphorylation, the inhibitor had no effect on filopodia. However, it rescued filopodia from PMA‐initiated destruction. This suggested that the PKC targeted by the inhibitor was activated by PMA, but the insertion of PKCI into the active site prevented the enzyme from binding its usual substrates.

Although these results suggest that the cPKCs may act to destroy filopodia following PMA, they do not prove it, because the α/β pseudosubstrate sequence may also bind nPKCs. It is known that the PKC ɛ pseudosubstrate competed with the corresponding PKC α sequence for binding partners in vitro (Liao, Hyatt, Chapline, & Jaken, 1994). Substrate specificity of the PKC isoforms is rather lax (Nishikawa, Toker, Johannes, Songyang, & Cantley, 1997). This lack of specificity would permit the PKC α pseudosubstrate to dock in the nPKC active site (Wu‐Zhang and Newton, 2013). Rescue was unaffected by inclusion of C2‐ceramide (Figure 5a). With a still lower (4 µM) concentration, the rescue effect was unchanged (data not shown). We attempted to inhibit the effect of nPKCs selectively by applying a myristoylated EGFR BP matching the specific sequence for PKC η substrates (Nishikawa et al., 1997). Concentrations representing the IC50 were toxic, however, and the results could not readily be interpreted.

### 2.4 Protein tyrosine phosphatase (PTP) inhibitors reproduce both patterns—Steady‐state elevation and filopodia rescue

It has long been known that PKC is associated with a phosphotyrosine‐containing protein that is a binding partner of integrin in filopodia (Wu, Wang, Mason, & Goldberg, 1996). Moreover, a similar substrate was thought to reside upstream of Rho GTPase where it could regulate focal adhesion assembly (Schoenwaelder and Burridge, 1999). Although neither the kinase responsible for phosphorylation nor the substrate is known, c‐Src (Rous sarcoma virus nonreceptor tyrosine kinase) may be a substrate of PKC (Thuringer et al., 2010). Thus, it is possible that PKC regulates filopodia upstream of c‐Src. In preliminary work, we found that phenylarsine oxide (PAO), a compound that modifies thiol groups, hence inactivating any PTPs with the XCysXXCysX motif, enhanced filopodia (De, 2015). PAO has many effects on the cytoskeleton, and in some cases affects RhoA GTPase, which has vicinal cysteines within the guanine‐nucleotide‐binding region and the phosphohydrolase active site (Gerhard, John, Aktories, & Just, 2003).

Taking advantage of the fact that there are new PTP inhibitors with K_m_ values several orders of magnitude lower than the PKC inhibitors, we undertook to determine whether a PTP dephosphorylated the hypothetic binding partner of PKC. Highly selective PTP inhibitors were used to identify candidate PTPs. When inhibitors of PTP1B, T‐cell PTP (TC‐PTP), PTP from megakaryocyte‐2 (PTP‐MEG2), and density‐enhanced phosphatase (DEP‐1) were used, two patterns emerged. PTP1B, PTP‐MEG2, and DEP‐1 enhanced filopodia in samples without PMA but had little effect in PMA‐treated samples. TC‐PTP inhibitor rescued filopodia from PKC‐initiated destruction, however, while decreasing the levels of filopodia in cells not treated with PMA. At concentrations above the *K*_i_, filopodia coverage in PMA‐treated samples was elevated 50% above the PMA‐treated control (Figure 5b). This showed that TC‐PTP (or another PTP affected by the same inhibitor) had destructive effects on filopodia like those of PKC ɛ, which were counteracted by the inhibitor. Another PTP might be destructive to filopodia formation or maintenance in the steady‐state, as indicated by the tendency of PTP‐MEG2 inhibitor to elevate filopodia at both concentrations (Figure 5b). This PTP was unlikely to be TC‐PTP itself, because TC‐PTP favored filopodia formation or maintenance under steady‐state conditions.

### 2.5 Comparison of PKC ɛ and cPKCs with respect to PMA‐stimulated turnover

**Figure 6:**
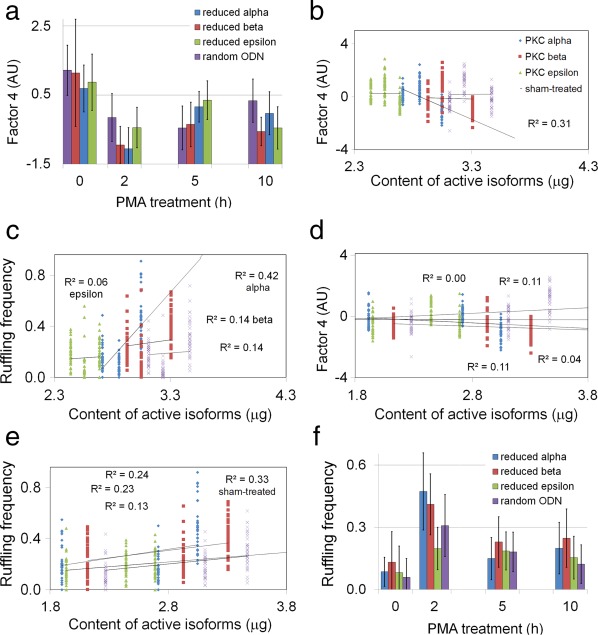
Filopodia prevalence in 1000W populations treated with antisense ODNs against PKC α, β, or ɛ. The control was treated with a random sequence ODN. (a) Mean factor 4 values for samples treated with knockdown reagent followed by PMA. (b) Individual cells' factor 4 values for 0–5 h times against summed content of PKCs in PMA‐treated cells. Coefficient of determination (*R* ^2^) is 0.01 or less for all knockdown samples except that with reduced PKC α, where *R* ^2^ was 0.31. (c) Individual cells' ruffling frequency for 0–5 h times against summed content of PKCs in PMA‐treated cells. The *R* ^2^ was low for all samples except that with reduced PKC α. (d) Individual cells' factor 4 values for 2–10 h times against summed content of PKCs in PMA‐treated cells. (e) Individual cells' ruffling frequency for 2–10 h times against summed content of PKCs in PMA‐treated cells. The *R* ^2^ was low for all samples except the sham‐treated control, where the *R* ^2^ value was 0.33. (f) Mean ruffling frequency for samples treated with ODN knockdown reagent followed by PMA. Bars represent ± 1 standard deviation [Color figure can be viewed at wileyonlinelibrary.com]

The above data suggested that PMA activates and translocates the PKC ɛ isoform to a membrane where it phosphorylates a substrate that destroys filopodia. The PKCI effect, however, suggested that PKC ɛ may not be the sole isoform responding to PMA (Figure 5A). We compared the possibility of parallel effects of PKC α/β with those of PKC ɛ by reducing the levels of these isoforms selectively and then analyzing filopodia prevalence. Because PKC molecules had a long half‐life under steady‐state conditions, it was impossible to eliminate them completely by transcriptional knockdown. Nevertheless, the effects of knocking down one isoform could be modeled to good effect, as long as the amounts of the other isoforms in the re‐balanced mixture were known (Table 1, Figures S3 and S4 in Supporting Information). We measured filopodia by an unbiased method in order to better detect the differences (see Materials and Methods). In the absence of PMA, the knockdown of any one isoform had little effect on the cell phenotype. After a 2‐h exposure to PMA, however, the filopodia prevalence in the PKC α knockdown sample was lower than in either the control or PKC ɛ knockdown samples (Figure 6a). The differences were statistically significant, as shown by the designation of the sample means by different letters on Table 2. With PKC β knockdown, the filopodia prevalence was significantly lower than the control but was indistinguishable from the value for PKC ɛ knockdown (Table 2). This suggested that PKC α protected filopodia from destruction by the PKCs whose content was left unchanged by treatments that knocked down PKC α transcription. Thus, it was unlikely that PKC α participated in promoter‐stimulated turnover. To confirm that the procedure was effective, the quantities of PKC α and ɛ were determined after knockdown of the respective isoforms (Table 3, Supporting Information Figure S2D,F).

**Table 1:** Content of PKC isoforms in 1000W cells at intervals after PMA treatment^a^

Isoform	Time after PMA treatment (h)					
	0	0.5	2	5	10	15
PKC α	0.600	0.917	0.846	0.797	0.740	0.533
PKC β	0.361	0.376	0.322	0.364	0.305	0.362
PKC γ	0.804	0.706	0.739	0.608	0.555	0.394
PKC δ	0.550	0.495	0.634	0.610	0.505	0.560
PKC η	0.634	0.575	0.621	0.680	0.716	0.662
PKC ɛ	1.46	1.25	1.56	1.34	0.669	0.491

Content in units of µg PKC per 150 µg of lysate protein.

**Table 2:** Classification of samples of Figure 6 by factor 4 values after transcriptional knockdown followed by PMA^a^

Tukey grouping							Mean	*N*	Combination of agents
A							1.22	31	Sham‐treated (0 h)
A							1.14	31	PKC β (0 h)
A							0.870	35	PKC ɛ (0 h)
A	B						0.689	37	PKC α (0 h)
	B	C					0.348	32	PKC ɛ (5 h)
	B	C					0.334	32	Sham‐treated (10 h)
	B	C	D				0.155	35	PKC α (5 h)
		C	D	E			−0.030	34	PKC α (10 h)
		C	D	E			−0.153	34	Sham‐treated (2 h)
			D	E	F		−0.353	34	PKC β KD (5 h)
			D	E	F	G	−0.445	33	PKC ɛ KD (2 h)
			D	E	F	G	−0.454	32	Sham‐treated (5 h)
			D	E	F	G	−0.456	34	PKC ɛ KD (10 h)
				E	F	G	−0.557	36	PKC β (10 h)
					F	G	−0.945	36	PKC β KD (2 h)
						G	−1.06	32	PKC α KD (2 h)

a Rows designated by the same letter are indistinguishable at level of confidence *P* < 0.05.

**Table 3:** PKC content remaining after treatment by BP interference or transcriptional knockdown with or without PMA treatment for 10 h^a^

Treatment	Isoform targeted			
	PKCα		PKCɛ	
	Knockdown	Irrelevant protein	Knockdown + 10 h PMA	Control + 10 h PMA
None	100%	N.A.^b^	N.A.	46%
ODN	43%	N.A.	22%	47%
siRNA	N.A.	N.A.	21%	53%
BP	80%	100%	N.A.	N.A.

a Percent of zero‐time value from Table 1 (µg PKC per 120 µg of lysate protein).

b N.A.= not applicable.

To visualize these interactions, the unbiased measure of filopodia (factor 4) was regressed against the content of the remaining isoforms in samples selectively reduced in PKC α, β, or ɛ. The content was based on the estimated content of all known PKCs in 1000W cells (Table 1). There was no evidence that PKC δ and η were ever activated by PMA, so they were omitted from the model. The coefficient of determination (*R* ^2^) was 0.01 or less for all knockdown samples except PKC α, where *R* ^2^ was 0.31. This meant that 31% of the variation in factor 4 values can be attributed to the size of the residual PKC pool. Therefore, the knockdown of PKC α content linked the mass of the remaining PKCs to the destruction of filopodia (Figure 6b). This effect may be indirect, however. For example, PKC α knockdown may merely decrease the number of molecules competing with other PKCs for PMA, or decrease the competition with other PKCs for occupancy of a scaffold.

The above result was investigated further to determine whether the resistance to filopodia destruction was related to ruffling activity, which was known to be inversely correlated with factor 4 (Heckman et al., 2012). By measuring ruffling frequency in the same cells as shown in Figure 6a,b, we found that the decrease in PKC α content caused a marked activation of ruffling. The *R* ^2^ for PKC α‐depleted samples was 0.42. The positive slope of this regression curve indicated that 42% of the increased ruffling could be attributed to an increase in the content of residual PKCs, which consisted mainly of the PKC ɛ and γ isoforms (Table 1). Therefore, PKC α conserved filopodia against PMA‐stimulated destruction. This effect may be indirect, e.g. by counteracting the stimulation of ruffling by ɛ and γ isoforms or other PMA receptors. Because the remaining PKCs largely consisted of ɛ and γ, and both were activated by PMA, it is probable that one or both enhanced ruffling. As the R^2^ levels for the other isoforms were <0.15, it was unlikely that any other PKCs, even PKC β, acted like PKC α (Figure 6c). PKC β was at the lowest level of any isoform in 1000W cells (Table 1). Thus, the data suggested that PKC α antagonized the removal of filopodia by one or more of the other PKC isoforms, probably by PKC ɛ.

Although their levels rebounded as the PKC content in the pool declined after 2 h of PMA treatment, filopodia never regained the prevalence observed at time zero (Figure 6a). Depletion of either PKC α or ɛ from the pool of PKC molecules caused a significant elevation in filopodia prevalence at 5 h (Table 2). This did not persist over longer times, however, as factor 4 values for samples between 2 and 10 h uniformly showed *R* ^2^ values <0.12 (Figure 6d). Although fluctuations in filopodia prevalence were independent of the size of PKC pool at later times after PMA exposure, ruffling frequency continued to show a positive relationship to PKC content. Ruffling frequency showed *R* ^2^ values greater than 0.2 (except for the epsilon knockdown) and positive slope. For the sham‐treated control, 33% of the increased ruffling could be attributed to differences in PKC content (Figure 6e). As PKC ɛ and PKC γ were known to be activated by PMA exposure (see Supporting Information Figure S2), they could continue to stimulate ruffling. Indeed, PKC ɛ knockdown decreased ruffling throughout the acute phase of ruffling stimulation (Figure 6f). The analysis was consistent with an interpretation that PKC ɛ activation was responsible for most of the filopodia destruction, and it destroyed filopodia by enhancing ruffling activity.

## 3 DISCUSSION

### 3.1 Two mechanisms for filopodia turnover

The findings in the current research show that one or more PKCs mediate filopodia destruction downstream of PMA. There are a number of other phorbol ester‐binding proteins, e.g. chimaerins, RasGRPs, Munc13, and DAG kinase (Brose and Rosenmund, 2002), which could not be entirely ruled out as true mediators of tumor promotion because the mechanism of tumor promotion is not completely understood. The finding that filopodia can be rescued from the effect of PMA by a domain of PKC ɛ, however, implicates PKC in filopodia dissolution and thereby in tumor promotion. The current results suggest that most or all PKC isoforms favor turnover of filopodia. There are two mechanisms of turnover, however. The prototypical PKC ɛ BP agent “rescues” filopodia from promoter‐stimulated destruction, indicating that one mechanism is related to treatment with tumor promoter and therefore of signaling from phospholipase C. The second mechanism affects the steady‐state level of filopodia. The former was of interest because of PKC ɛ's relationship to malignancy. In the classical model system for tumor promotion, transgenic mice overexpressing PKC ɛ showed suppression of benign tumors but 6‐fold elevation in carcinomas (Jansen et al., 2001). Of all isoforms of PKC, PKC ɛ is most frequently found as an oncogene in various types of cancer. The relationship between PKC ɛ and malignancy is discussed in recent reviews (Garg et al., 2014; Urtreger, Kazanietz, & Joffe, 2012). In contrast, PKC η acts as a tumor suppressor gene in the skin (Kashiwagi, Ohba, Chida, & Kuroki, 2002).

One mechanism by which the BP rescues filopodia may have to do with the capability of PKC ɛ to enhance PDK1 autophosphorylation (Garczarczyk et al., 2009). PKC ɛ's hydrophobic motif must be phosphorylated, however, in order for PDK1 to bind (see Introduction). Thus, competition for this site by PKC ɛ BP may inhibit phosphorylation at the hydrophobic motif and inhibit PDK1 docking on PKC ɛ. PDK1 phosphorylates many AGC kinases (PKA (protein kinase A)/PKG (protein kinase G)/PKC (protein kinase C)‐kinase) in their activation loop (Mora, Komander, van Aalten, & Alessi, 2004). By inhibiting the interaction of PKC ɛ with PDK1, the PKC ɛ BP could depress the rate of AGC kinase activation in general. Whereas there is evidence that PKC η also regulates PDK1 (Bär, Rommelaere, & Nüesch, 2015), we note that there is no evidence that it was activated by PMA in these experiments. It should be noted that the PKC isoforms are generally downstream of PDK1 and so would be inhibited along with other AGC kinases (Le Good et al., 1998). Although it is unlikely that a decrease in AGC kinases overall could be responsible for the “rescue” effect, which was specific to a kinase or kinases activated by PMA, it is possible that the rescue was caused by BP‐mediated repression of PKC ɛ maturation. To date, we have not been able to implicate another kinase in addition to PKC ɛ in filopodia destruction. Another possible mechanism by which the PKC ɛ BP conserves filopodia is that, if the hydrophobic BP becomes phosphorylated, it competes for the site where PKC ɛ is localized at the Golgi region, which requires phosphorylation at the hydrophobic motif (Xu et al., 2007).

The above mechanisms appeared specific to PKC ɛ, as filopodia were not rescued by the BPs for other nPKCs found in 1000W cells. The contrast between the isoforms is especially noteworthy in regard to PKC η. PKC η, like PKC ɛ, is often localized to the Golgi apparatus. The nPKCs are capable of phosphorylating PKD at the activation loop (Brändlin et al., 2002b; Döppler and Storz, 2007; Waldron, Iglesias, & Rozengurt, 1999; Waldron and Rozengurt, 2003; Wang et al., 2009). PKC η acts downstream of a β1γ2 or β3γ2 heterodimer from G protein‐coupled receptor and forms a complex which drives the activation of PKD and the generation of complexes between PKC and PKD localized to the TGN membrane (Añel and Malhotra, 2005). It is thought that both PKC η and ɛ can phosphorylate residues in the activation loop of PKD, driving activation in some cell types. However, a PDK1/PKC η/PKD complex can be formed that exerts negative regulation on PKC η (Brändlin, Eiseler, Salowsky, & Johannes, 2002a). The PKC η/PKD complex is unique among the nPKCs in two ways: (1) it is essential for Golgi integrity (Añel and Malhotra, 2005) and (2) it is localized to a scaffold by A‐kinase anchoring protein, AKAP‐Lbc (Carnegie, Smith, McConnachie, Langeberg, & Scott, 2004). Moreover, PKC η and PKC ɛ work in opposition on some of the functions done by PKD at the trans‐Golgi network, including cargo packaging, constricting the emerging vesicle bud, membrane fission, and recruiting the correct molecular motors to direct transport carriers. Further investigation must be done to pinpoint the differences. The AKAP‐Lbc scaffold is not at the Golgi or trans‐Golgi network, so it may provide sites for complex docking that compete with those at the Golgi compartments (Añel and Malhotra, 2005).

Effects on steady‐state processes were revealed by the BPs against PKC α and PKC η. These agents enhanced filopodia in the absence of PMA but failed to rescue them from PKC‐initiated destruction. In previous work, the extent of degradation of an isoform in cells treated with a tumor promoter has been considered a surrogate measure of activity (see Supporting Information Figure S2). Although the lack of degradation (Table 1, Supporting Information Figures S3 and S4) implied a lack of activation of PKC α and η, it was not possible to measure the activity of PKC and get direct confirmation in 1000W cells. Activity was not retained in lysates prepared under denaturing conditions, and the full‐length protein could not be recovered from preparations made under non‐denaturing conditions. As the removal of the N‐terminal regulatory portion causes constitutive activation, measurements of the activity on these gels would be equivalent to the mass. If there is negative regulation of PKC η by PKC η, as mentioned above, the inhibition of PKC η/PDK1 complex formation could limit the activation of PKD. The BP targeting PKC η may then affect downstream pathways through its effect on PKD. This kind of regulatory complexity is not unique to the AGC kinases, as several kinases are known to be both upstream and downstream of another kinase.

The mechanism of action of the PKC α BP remains unknown. It is unlikely that PKC α or β was hyperactivated by the promoter treatment, as they are activated synergistically by diacylglycerol and calcium (Liu and Heckman, 1998), and the latter is generally at low concentrations in the cell. The role of PKC α in opposition to PKC ɛ also remains unknown. The role of PKC α may be passive, i.e., it may compete for a scaffold or receptor that is also occupied by PKC ɛ. There is no evidence in these studies that the PKC α BP targets the same cellular trafficking functions as that against PKC η. The effects of the PKC α BP, however, suggested destabilization of the folded structure, which would be expected in the absence of a stabilizing phosphorylation at the hydrophobic motif (Gould and Newton, 2008; Newton, 2010). The lack of phosphate at the hydrophobic site prevents PKC maturation to the competent state and increases its rate of degradation (Gysin and Imber, 1996). Thus, the BP may work by decreasing the amount of competent protein, as well as increasing its turnover. Our determinations of PKC mass recovery with phospho‐PKCα (Ser657/Tyr658) antibody with and without PMA treatment suggested that PMA caused an increase in competent molecules. This may have contributed to the effect by which the alpha isozyme counteracted the PKC ɛ‐mediated destruction of filopodia in PMA‐treated cells (Figure 6a). Neither activity nor competency, however, can be predicted from phosphorylation states, as mentioned previously by other researchers (Freeley et al., 2011; Kunkel and Newton 2015).

To gain additional information as to what other PKCs responded to PMA in the system, we used a myristoylated BP inhibitor of cPKCs. The inhibitor rescued filopodia from PMA‐initiated destruction but had little effect on filopodia in the steady state. As mentioned above, there was no way of excluding that it inhibited PKC ɛ. C2‐ceramide treatment did not affect the rescue of filopodia by the PKC α/β inhibitor. C2‐ceramide selectively inhibits the translocation of cPKCs to membranes (Jones and Murray, 1995) and stimulates a serine/threonine phosphatase that dephosphorylates residues at the activation loop (Kitatani, Idkowiak‐Baldys, & Hannun, 2007), so the lack of effect in the experiments could be because the inhibitor actually targeted PKC ɛ. Although PKC γ was known to be activated following treatment with nanomolar concentrations of PMA (Li et al., 2006), PKC γ BP appeared to have little effect on filopodia.

### 3.2 Tyrosine kinase substrate associated with PKC

As mentioned above, there is previous evidence for a tyrosine kinase substrate closely associated with focal contacts. TC‐PTP binds to and is activated by the integrin α1 subunit, possibly at the focal contact (Mattila et al., 2005). Because the proto‐oncogene, c‐Src, and PKC are transient components of the focal contacts, the kinase may be c‐Src. If so, several candidate substrates exist. One, p130Cas (Crk‐associated substrate), is both a scaffold for c‐Src and a substrate whose many phosphorylation sites are subject to processive phosphorylation (Pellicena and Miller, 2001). A second, FAK (focal adhesion kinase), is required for the turnover of focal adhesion sites (Westhoff, Serrels, Fincham, Frame, & Carragher, 2004). Other probable substrates of c‐Src, e.g., PKL/GIT2 (paxillin kinase linker/G‐protein coupled receptor kinase‐interacting protein) or paxillin, may be candidates for the unknown phosphorylated component of filopodial focal contacts. Unfortunately, no specific inhibitor of c‐Src was available. Experiments with highly selective PTP inhibitors confirmed the existence of two patterns. Thus, PTP inhibitors could be grouped into classes. A number of them enhanced filopodia in the steady‐state without rescuing them from PKC‐mediated destruction. The second class, exemplified by TC‐PTP, replicated the effect of PKC ɛ BP.

### 3.3 Role of promoter‐specific turnover in cell motility

The data suggested that, in the epithelial cells employed here, PKC ɛ is activated by PMA and stimulates ruffling which, in turn, removes filopodia. Moreover, the data showed that, at the levels normally present in cytoplasm, PKC α suppressed ruffling and thereby inhibited the destruction of filopodia by PMA. In turning over filopodia, PKC ɛ probably circumvents the role of both PKC α and η. Currently, we have little understanding of how this promoter‐specific turnover contributes to carcinogenesis and tumor promotion. Nevertheless, as cells lose sensors during their conversion into cancer cells, they may have a weaker response to contact and less directional persistence, or they may treat all surfaces as unfavorable for attachment. Our finding that PKC α can suppress ruffling and thereby affect promoter‐specific turnover of filopodia reinforces the need for investigating PKCs as a system. As *R* ^2^ values around 0.3 represent minor effects, the suppression of ruffling observed is small. Nevertheless, it is clear that there is some opposition in the cells between PKC α and PKC ɛ. PKC α can act as a tumor suppressor or an oncogene, depending on the context, so the same duality has been found for its in vivo activity (Griner and Kazanietz, 2007).

In the context of signaling, what benefit would accrue to a cell if it ensured the long‐term suppression of filopodia? It may be a mnemonic device. In previous work, we showed that cells require filopodia in order to orient themselves on a haptotactic gradient (Amarachintha et al., 2015). Cells may activate PKC ɛ selectively on the less adhesive margin of the cell and thereby reduce the prevalence of filopodia. When a cell meets a surface that is unfavorable for attachment, it may retract filopodia through the promoter‐specific turnover mechanism while setting a marker to “remember” the direction of unfavorable contact. In nature, the marker is probably the sustained formation of diacylglcerol from phosphatidylcholine via PLD and generation of phosphatidic acid, which are required to maintain PKC in the membrane (Lopez‐Andreo, Gomez‐Fernandez, & Corbalan‐Garcia, 2003). This mechanism may be invoked during development of the nervous system, accounting for the importance of PKC in neural guidance (Bonsall and Rehder, 1999; Cheng, Mao, & Rehder, 2000).

## 4 MATERIALS AND METHODS

### 4.1 Cell culture

The 1000W cell line was derived as an outgrowth from a heterotopic tracheal transplant in a F344 inbred rat, following exposure to 7,12‐dimethylbenz(a)anthracene (Marchok, Rhoton, & Nettesheim, 1978). The epithelial cells were cultured in Waymouth MB752/1 medium (Sigma–Aldrich, St. Louis, MO) supplemented with 10% fetal bovine serum (Hyclone, UT or Atlanta Biologicals, GA), as previously described (Amarachintha et al., 2015; Heckman, Plummer, & Runyeon, 1996). The line was tested periodically and found to be free of contamination with Mycoplasma. The 1000‐W cells were tested periodically for tumorigenicity in immune‐suppressed, syngeneic animals. After 65 weeks in culture, they were positive for tumorigenicity, suggesting that long‐term culture in vitro has an effect similar to tumor promotion. Cells used in these experiments were at 35–45 weeks of in vitro culture. For experiments, glass coverslips were coated with germanium for use as substrates (see Preparation of substrates). Cells were subcultured at a density of 250,000 per 35‐mm dish and incubated overnight to allow them to adhere to the substrates. Then, they were treated with reagents as described below (see Treatment with BPs, small inhibitory RNAs (siRNAs), and ODNs).

### 4.2 PKC content determination

To determine the content of PKC isoforms, we lysed cells from 60‐mm dishes using either a denaturing solution (2.3% SDS, 10% glycerol, 5% β‐mercaptoethanol, and 62.5 mM *tris*(hydroxymethyl)aminomethane (Tris), pH 6.8) or a non‐denaturing solution (see below). Protease inhibitor cocktail (Roche Applied Science, Mannheim, Germany) was used in the lysate solution according to manufacturer's recommendations. Total protein content of lysates varied from 900 to 2400 µg, and each time point in the time course of PMA treatment was represented by at least five replicate cultures. Preliminary studies showed that PKC content did not vary with cell density. To recover specific isoforms, we made up an aliquot of lysate in a mixture of deionized water and 2× immunoprecipitation buffer (2% Triton X‐100, 300 mM NaCl, 2 mM ethylenediaminetetraacetic acid (EDTA), 2 mM ethylene glycol‐bis(β‐aminoethyl ether)‐*N*,*N*,*N*′,*N*′‐tetraacetic acid (EGTA), 400 µM sodium orthovanadate, 400 µM phenylmethylsulfonyl fluoride, 1% NP‐40, and 20 mM Tris, pH 7.4) and an aliquot of 3 µg antibody was added. All of the antibodies were directed against the portions of PKC sequence as shown in Table 4. The antigen‐antibody complex was recovered on protein A‐agarose beads (Invitrogen, Carlsbad, CA). Controls were run by addition of agarose beads to the immunoprecipitation mixture in the absence of primary antibody. No protein bands were observed in the controls. Likewise, when the peptide used as the antigen was included as a blocking peptide in the immunoprecipitation mixture, recovery was completely abolished. The bands were quantified by using the gel analysis module of ImageJ to calculate OD after background subtraction.

**Table 4:** Reagents used

Blocking peptides for PKC		
Target	Amino acid sequence	Residues
PKC α	EGFSYVNPQFVH	654–665
PKC δ	KGFSFVNPKYEQ	659–670
PKC η	RNFSYVSPELQP	672–683
PKC ε	KGFSYFGEDLMP	726–737
MARCKS	SFKKSFKLSGFS	152–163
MARCKS	FSFKKSKKEAGE	162–173
PKC‐derived antigens		
PKC α hinge (Life Tech.)	AGNKVISPSEDRKQ	313–326
PKC α (R&D)	MDHTEK….TVRDAKN	153–182
PKC α (USBiological)	VHPILQSAV	664–672
phospho‐PKC α (EMD)	…FEGF(p)S(p)YVNPQ…^a^	Region around phospho (Ser657/Tyr658)
PKC β (Life Tech.)	GPKTPEEKTANTISKFDC	313–329
PKC γ (Life Tech.)	NYPLELYERVRTG	306–318
PKC δ (Life Tech.)	SFVNPKYEEFLE	664–675
PKC ε (Life Tech., EMD)	KGFSYFGEDLMP	726–737
PKC η (Transduction Lab.)	NCGVN…….FHIQK	181–334

a Exact domain not provided by the supplier.

Because there was a phosphorylation site in PKC α at S319 (Figure 4i), and occupancy of this site could lead to poor recovery of the isoform, we also used rabbit antibodies directed against antigens representing the phosphorylated hydrophobic motif, phospho‐PKC α (Ser657/Tyr658), and the extreme C‐terminal, 664–672 (Table 4). To conserve phosphorylation sites, we recovered the cells in a lysis solution containing 1% NP‐40, 100 mM NaCl, 20 mM Tris, 1 mM EDTA, 1 mM EGTA, 2.5 mM sodium pyrophosphate, 1 mM β‐glycerophosphate, and 1 mM sodium orthovanadate (pH 7.8). Except in rare instances, the manufacturers did not test cross‐reactivity with the phosphorylated and dephosphorylated sequences of other PKC isoforms, but the homology around (phospho‐PKC α(Ser657/Tyr658)) is such that PKC β cross‐reactivity is likely. All the PKC isoforms have phosphorylation sites at adjacent Ser and Tyr residues, although the phospho‐Tyr form of the protein is rarely recovered (http://www.phosphosite.org).

### 4.3 Preparation of substrates

Coverslips of 25‐mm diameter and thickness #1 (Electron Microscopy Sciences, Hatfield, PA) were rendered molecularly clean, air dried, and coated in a Denton BTT‐IV high vacuum evaporator. About 20 mg of Ge (Structure Probe, West Chester, PA) was placed in a tungsten basket and evaporated at a resistive current of 9 Amp for 2 min in a vacuum of 2 × 10^−5^ Torr. Coverslips were sterilized by ultraviolet light irradiation before being used as substrates.

### 4.4 Treatment with BPs, small inhibitory RNAs (siRNAs), and ODNs

We designed peptide sequences corresponding to the phosphorylation sites within the hydrophobic sequence of protein kinase Cs (Table 4). We also investigated MARCKS as a candidate for regulating filopodia. To this end, BPs were also designed to block the three sites, 152, 153, and 163, phosphorylated in the effector domain (Heemskerk, Chen, & Huang, 1993). Peptides were delivered into the cells with BioPORTER reagent (Genlantis, San Diego, CA), a lipid formulation that facilitates the transfer of peptides and proteins into the cytoplasm. It was previously found to be harmless to 1000W cells (Heckman et al., 2009). The culture medium was replaced with 1.2 mL of serum‐free medium, and then the BioPORTER‐peptide mixture was added. After incubation for 8 h, the serum concentration was restored to 8.75%, and the samples incubated for at least 21 h longer. One set of dishes was treated with PMA at a final concentration of 2 nM for 30 min. The duplicates were treated with solvent vehicle only. A fluorescein isothiocyanate (FITC)‐tagged antibody supplied as a control by the manufacturer was used in sham‐treated control dishes. Cells were collected for imaging by immunofluorescence, in order to visualize protein internalization [see (Amarachintha et al., 2015) for details].

For knockdown procedures, reagents were introduced using Lipofectamine 2000 (Invitrogen) according to the manufacturers' instructions. The procedures for introducing the reagents were the same as above. The amount of PKC left after knockdown was determined by SDS‐PAGE or dot blots, using antibodies against PKC α and PKC ɛ (Table 4). For dot blots, different amounts of antigen were spotted onto Osmonics nitrocellulose strips in 6‐µL aliquots. Dot blots were processed by a standard protocol (Kielkopf, Bauer, & Urbatsch, 2012).

### 4.5 Reagents

Peptides were synthesized with C‐terminal amidation by Peptide 2.0 (Chantilly, VA). PKC α/β myristoylated inhibitor (19–27) and EGFR fragment 651–658 were purchased from Calbiochem (Billerica, MA), and PKC‐zeta pseudosubstrate from Invitrogen. The myristoylated 19–27 inhibitor, PKCI, was previously reported to inhibit activation of PLD at a concentration of 20 µM and phosphorylation of MARCKS at 8 µM (Eichholtz, de Bont, de Widt, Liskamp, & Ploegh, 1993). PKD1 and PKD2 siRNAs were purchased from Origene (Rockville, MD). PKC γ siRNAs and PKC morpholino‐oligodeoxynucleotides were purchased from Qiagen (Germantown, MD) or Sigma (St. Louis, MO). The ODN sequences are as previously described (Li et al., 2006). PTP inhibitors were synthesized as previously described (Zhang et al., 2009). The inhibitory constants (K_i_) for the compounds shown, TC‐PTP and PTP‐MEG2, were 4 and 34 nM, respectively.

### 4.6 Fixation, microscopy, and analysis

Dishes containing Ge‐coated substrates were fixed for 10 min with warm (37°C) formaldehyde made up from paraformaldehyde in cytoskeletal buffer (pH 7.4). Samples were rinsed with phosphate‐buffered saline and stored refrigerated until analyzed.

Each substrate was labeled with a code number, mounted on a glass slide, and sealed with nail polish, then observed by phase contrast in a Zeiss Axiophot light microscope using a Plan‐Neofluar 100×/1.30 objective lens. Samples were imaged in a raster pattern, and all single cells with clearly visible edges were scored. Any cell with 25% or more of the edge rounded up was excluded from the analysis (De, 2015). Because the 1000W cells adhered tightly to the substrate, retraction fibers were rarely seen, and all the filopodia were adnate under conditions of the experiments. Occasionally, wavy filopodia which were thought to represent retracting filopodia were seen (Figure 3). The images of Figure 3 represent the full range of morphologies found in the samples. Filopodia of the 1000W cells never rose vertically from the substrate, nor did the agents used here ever cause them to loop back and adhere to the cell body through their tips. Structures were excluded from the analysis if they showed a decrease in actin bundling (Figure 3j) or were too large (Figure 3k). The standard for selecting filopodia was based on illustrations of cells rich in filopodia as defined by automated pattern processing and analysis software (Heckman et al., 2012). For each sample, counts were made by two or three independent observers.

It should be noted that the number of filopodia on the cell perimeter was subject to considerable variation in repeat experiments. This was due in part to varying conditions of attachment, spreading and growth of the cells, which cannot be precisely replicated in repeat experiments. A predictable pattern of decrease in filopodia occurs, however, as shown in Figure 1b. Thus, raw coverage could vary from ∼5% to 20%, and the data were typically presented as a ratio between the counts for each sample to baseline represented by sham‐treated control. The number of cells counted on each sample is shown in Supporting Information Table S1.

### 4.7 Unbiased classification of cell features

The above method used to quantify filopodia relied mainly on a subjective evaluation of the cell perimeter. A more general method of classifying cell features, based on the mathematical deconstruction of the cell into its native parts, was used in the transcriptional knockdown experiments, however. This enabled features to be recognized qualitatively and their prevalence in a sample to be evaluated on a quantitative basis. The initial classifications were derived from images acquired by Tolansky interference, which allowed information from three dimensions to be included in the data compilation (Heckman and Jamasbi, 1999). Each of the three lowermost interference contours was evaluated with respect to 34 geometrical variables, and each of these 102 variables was rendered dimensionless by dividing them by the value of a dimensioned variable, i.e., area, perimeter, or length of the major axis of the ellipse of concentration. By extracting principal components and computing the latent factors, i.e. theoretical variables that account for the covariance of variables in the primary variable dataset, we reduced the variable set to 20 factors. For samples collected in experiments, each primary variable was multiplied by a positive or negative loading constant to convert the values into the factor score. SAS software (SAS Institute, Cary, NC) was used together with software written by the laboratory (Heckman et al., 2009) for data conversion into factor values.

Valid solutions for the edge features could be obtained from images generated by scanning electron microscopy (Heckman et al., 2009) or phase microscopy (Amarachintha et al., 2015). Cells were prepared for scanning electron microscopy as previously described (Heckman, Kanagasundaram, Cayer, & Paige, 2007). By extracting the edge contour of each cell and processing the digital information as above, we could detect small differences and analyze features without relying on subjective criteria. In these experiments, the prevalence of filopodia (factor 4) was analyzed starting with samples imaged in a Hitachi S2700 scanning electron microscope. Edge contours were converted into digital format and contour extraction performed as previously described (Heckman et al., 2009). At least 30 cells were sampled for each experimental treatment.

The current method was compared with other methods of semi‐automated feature analysis. The other methods are designed to automate the analysis of features that are already known, and are mainly applied to neurons where the filopodia are large and concentrated in growth cones or dendrites. One such method (Hendricusdottir and Bergmann, 2014) requires the operator to manually identify the tip and base of the filopodium in one of a series of time‐lapse images. The direction of travel of the dendrite must also be determined manually. We note that another of these methods (Costantino et al., 2008) requires manual selection of a threshold for converting gray‐level to binary format. Manual setting of a derivative value for the Laplacian of two‐dimensional Gaussian for edge detection is also required. Finally, the efficacy of these methods must be confirmed by tracing the same structures manually or using other programs for tracking dynamic changes. The objective of these studies was to analyze dynamic changes in length, as well as vertical and lateral tilt of filopodia. These, as well as an additional technique designed to determine the presence of proteins along the length of the filopodium (Saha et al., 2016), are not suitable for data sets comprising thousands of filopodia. These methods were not congruent with the objective of the current studies, which was to understand prevalence in order to identify mechanisms for filopodia regulation. In the current research, subjective evaluations were used to identify agents that affected filopodia regulation. Completely unbiased feature identification was essential to identify morphological changes occurring in oncogenic transformation.

### 4.8 Statistics

Values for filopodia obtained by phase microscopy were tested and found to occupy a normal distribution. Calculations and statistical analysis were done using Excel, except for the Student's t‐test, which was done using the GraphPad online service. Statistical tests on samples processed by unbiased classification were done with SAS software (see Unbiased classification of cell features).

## Supporting information

Supporting Information Figure S1

Supporting Information Figure S2

Supporting Information Figure S3

Supporting Information Figure S4

Supporting Information Table S1
