# Supplementary figures and images for: The tumor promoter‐activated protein kinase Cs are a system for regulating filopodia
Source: Cytoskeleton (Hoboken). 2017 May 24;74(8):297–314. doi: 10.1002/cm.21373 (PMC5575509; doi:10.1002/cm.21373)

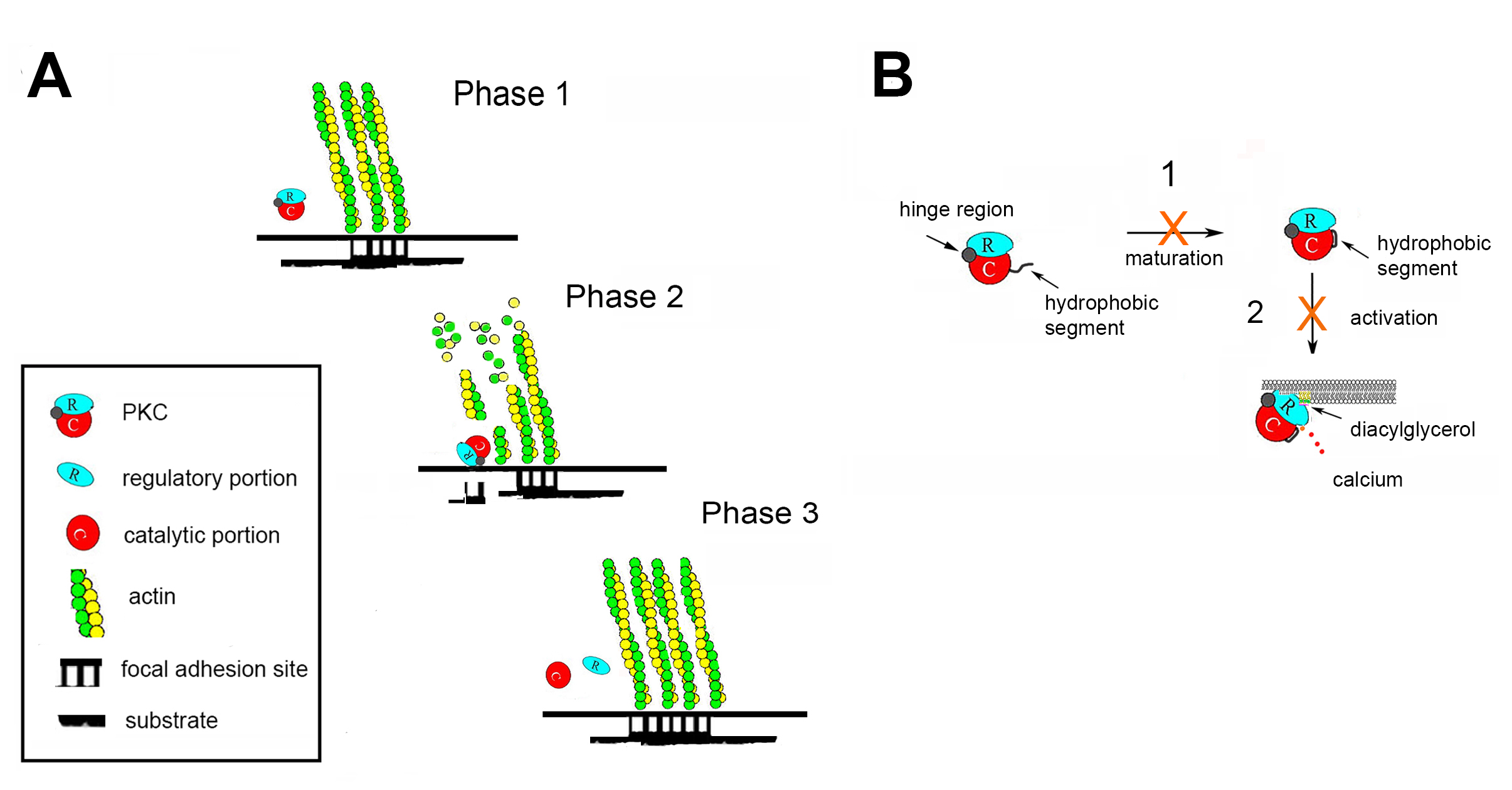

Supplement: Supplementary file 1 — Supporting Information Figure S1 [file CM-74-297-s001.jpg]

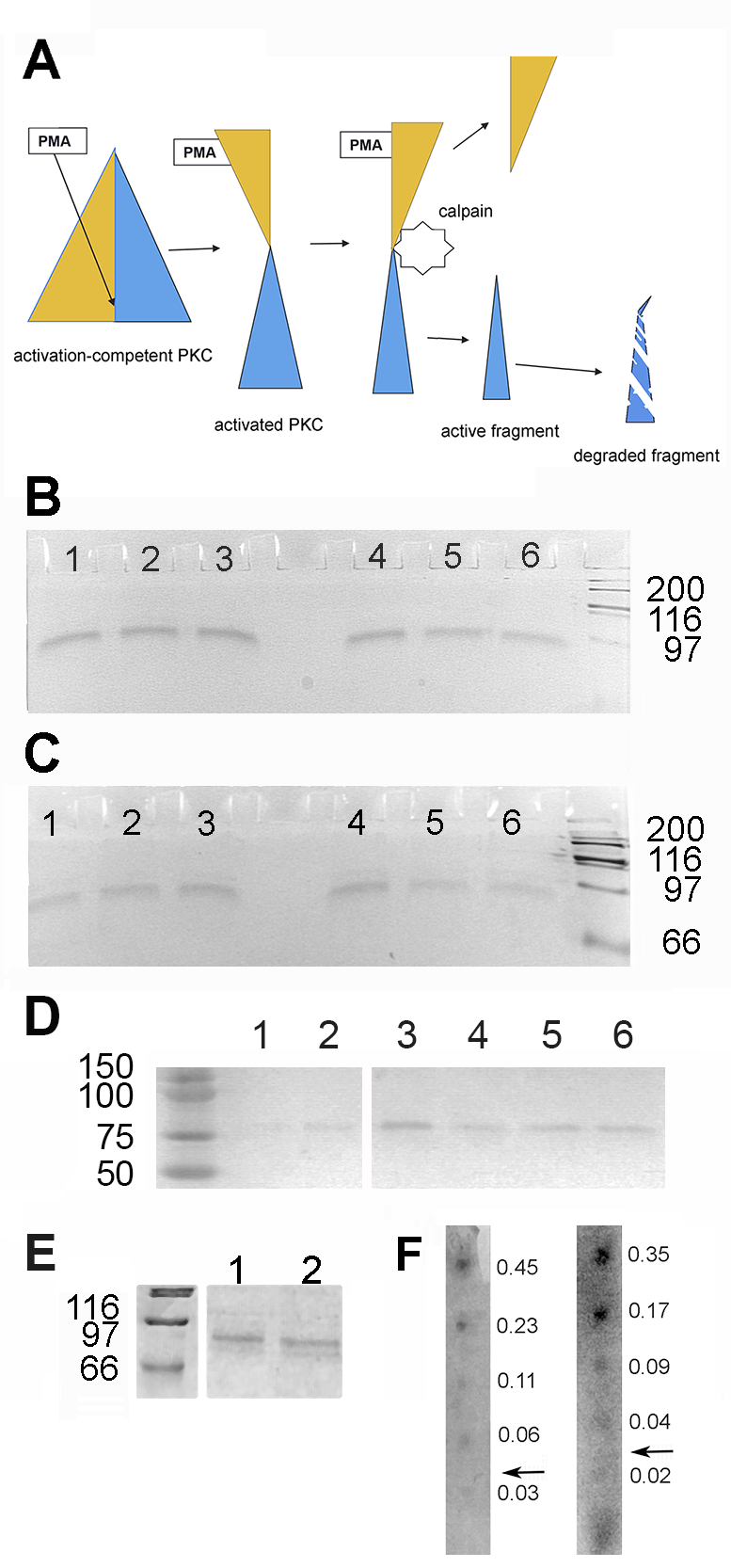

Supplement: Supplementary file 2 — Supporting Information Figure S2 [file CM-74-297-s002.tif]

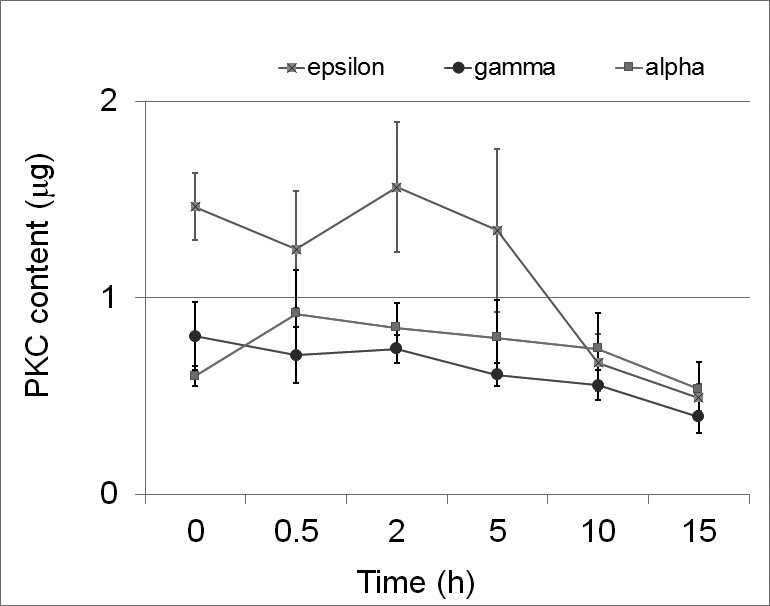

Supplement: Supplementary file 3 — Supporting Information Figure S3 [file CM-74-297-s003.tif]

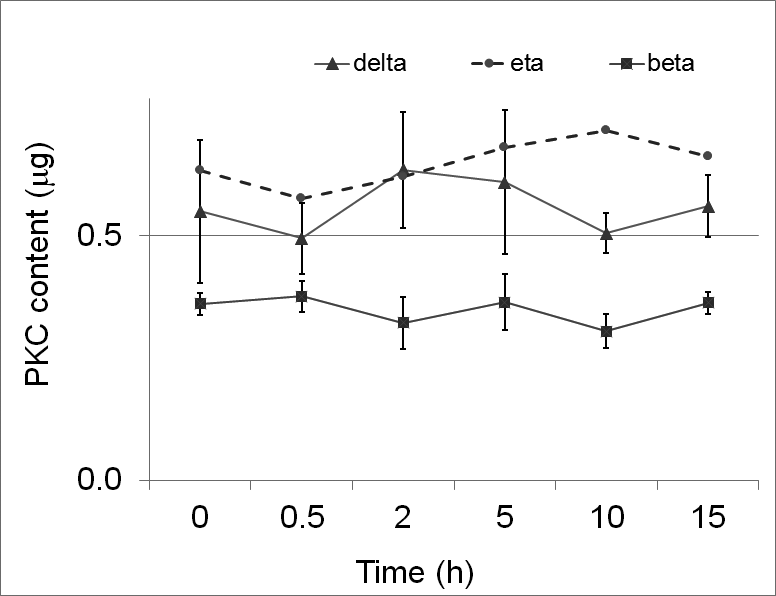

Supplement: Supplementary file 4 — Supporting Information Figure S4 [file CM-74-297-s004.tif]
