# Supplementary material for: The tumor promoter‐activated protein kinase Cs are a system for regulating filopodia
Source: Cytoskeleton (Hoboken). 2017 May 24;74(8):297–314. doi: 10.1002/cm.21373 (PMC5575509; doi:10.1002/cm.21373)
Supplement: Supplementary file 5 — Supporting Information Table S1 [file CM-74-297-s005.docx]

Table 5. Number of cells used in the main experimental studies, counted by different observers*

| Treatment | Number of samples | Obs. 1 | Obs. 2 | Obs. 3 | Mean cell number analyzed |
| --- | --- | --- | --- | --- | --- |
| Hydrophobic peptides |  |  |  |  |  |
| Experiment 1 | 7 | 966 | 408 | 333 | 244 |
| Experiment 3 | 12 | 1865 | 1127 | 411 | 284 |
| Experiment 4 | 14 | 867 | 1004 |  | 134 |
| Experiment 9 | 14 | 1215 | 1148 | 771 | 224 |
| Experiment 10 | 18 | 1639 | 1828 | 929 | 244 |
| Experiment 11 | 16 | 1494 | 1692 | 753 | 246 |
| Experiment 12 | 14 | 669 | 816 | 465 | 139 |
| PTP inhibitors |  |  |  |  |  |
| Experiment A | 14 | 1128 | 1482 | 475 | 220 |
| Experiment B | 14 | 1953 | 1603 | 901 | 318 |
| Experiment C | 14 | 1777 | 1232 |  | 215 |
| Experiment D | 12 | 1358 | 1129 | 620 | 259 |
| Myr19-27, EGFR and PKC ζ, and ROS inhibitors | | |  |  |  |
| Experiment E | 9 | 610 | 910 |  | 169 |
| Experiment F | 6 | 412 | 501 |  | 152 |
| Experiment G | 5 | 339 | 533 |  | 174 |
| Experiment H | 5 | 172 | 220 |  | 78 |
| Experiment I | 8 | 1052 | 1240 | 996 | 287 |
| Experiment J | 10 | 270 | 726 |  | 100 |
| Experiment K | 12 | 652 | 804 |  | 121 |

*observers (Obs.)
